# Gold Nanocylinders on Gold Film as a Multi-Spectral SERS Substrate

**DOI:** 10.3390/nano10050927

**Published:** 2020-05-11

**Authors:** Wafa Safar, Médéric Lequeux, Jeanne Solard, Alexis P. A. Fischer, Nordin Felidj, Pietro Giuseppe Gucciardi, Mathieu Edely, Marc Lamy de la Chapelle

**Affiliations:** 1 Institut des Molécules et Matériaux du Mans (IMMM - UMR CNRS 6283), Université du Mans, Avenue Olivier Messiaen, 72085 Le Mans CEDEX 9, France; wafasafar@hotmail.com (W.S.); mathieu.edely@univ-lemans.fr (M.E.); 2 Laboratoire CSPBAT, Université Sorbonne Paris Nord, CNRS, (UMR 7244), 74 rue Marcel Cachin, 93017 Bobigny, France; medlequeux@gmail.com; 3 Laboratoire de Physique des Lasers, Université Sorbonne Paris Nord, CNRS, (UMR 7538), 99 av. JB Clément, 93450 Villetaneuse, France; jeanne.solard@univ-paris13.fr (J.S.); fischer@univ-paris13.fr (A.P.A.F.); 4 Université de Paris, ITODYS, CNRS, UMR 7086, 15 rue J-A de Baïf, F-75013 Paris, France; nordin.felidj@univ-paris-diderot.fr; 5 CNR IPCF, Istituto per i Processi Chimico-Fisici, Viale F. Stagno D’Alcontres 37, I-98158 Messina, Italy; gucciardi@ipcf.cnr.it

**Keywords:** plasmon, SERS, optimization, multi-spectral substrate

## Abstract

The surface enhanced Raman scattering (SERS) efficiency of gold nanocylinders deposited on gold thin film is studied. Exploiting the specific plasmonic properties of such substrates, we determine the influence of the nanocylinder diameter and the film thickness on the SERS signal at three different excitation wavelengths (532, 638 and 785 nm). We demonstrate that the highest signal is reached for the highest diameter of 250 nm due to coupling between the nanocylinders and for the lowest thickness (20 nm) as the excited plasmon is created at the interface between the gold and glass substrate. Moreover, even if we show that the highest SERS efficiency is obtained for an excitation wavelength of 638 nm, a large SERS signal can be obtained at all excitation wavelengths and on a wide spectral range. We demonstrate that it can be related with the nature of the plasmon (propagative plasmon excited through the nanocylinder grating) and with its angular dependence (tuning of the plasmon position with the excitation angle). Such an effect allows the excitation of plasmon on nearly the whole visible range, and paves the way to multispectral SERS substrates.

## 1. Introduction

Surface enhanced Raman scattering (SERS) is a powerful tool for the identification and the quantification of chemical or biological species [1,2,3,4,5,6,7,8,9,10]. Although largely developed for the detection of analytes, a large number of sensors were proposed in the literature in a wide range of applications, e.g., for sensors [11], luminescence enhancement [12], emission color tuning [13], detection of biomarkers for disease diagnosis [14,15,16], detection of explosives for citizens security [17], of food contaminants for consumers safety [18,19] or of pollutants for the determination of environmental status [20,21,22].

Since the discovery of SERS [23,24], efforts have been made to produce structures that promote reliable and efficient signal enhancement. Two main types of SERS structures were developed: colloidal nanoparticles in solution [25,26,27,28] or deposited on a surface [29]. However, in both cases, the nanostructures geometrical parameters (size distribution) are often not well-controlled due to production limitations (random deposition, size distributions) and can lead to disordered samples.

In these cases, the presence of hot spots (highly localized area of intense electric field) may lead to significant enhancement but the SERS signal is not homogeneous and not reproducible [30] and depends on the experimental conditions and the measurement position in solution or on the substrate. In the context of sensor development and in order to provide unambiguous analyte quantification, the enhancement should be highly reproducible and it is necessary to optimize the SERS efficiency in relation with the plasmon excitation conditions [25]. For instance, the SERS signal is directly related with the excitation wavelength and Raman scattering wavenumber [25,31,32,33,34,35,36,37,38] or the coupling between nanostructures [39,40,41].

It is then of utmost importance to provide well-designed arrays to have a controlled enhancement coming from the nanostructures [42,43,44,45,46,47,48]. In this framework, SERS substrates composed of gold nanostructures deposited on gold film were recently proposed providing well controlled and reproducible SERS signal [49]. It has also been demonstrated that such substrates provide higher SERS efficiency, more homogeneous enhanced field distribution and directional SERS response compared to nanostructures on a dielectric surface [50,51,52].

The SERS signal on such substrate can be mainly explained by their specific plasmonic properties due to the coupling of propagative and localized surface plasmons inducing the excitation of hybrid modes [53,54]. For such substrates, previous studies have shown that the plasmonic properties are mainly controlled by two parameters: the gold film thickness and the nanostructure grating period (interparticle distance). As both geometrical parameters have strong influence on the excitation and the energy of the propagative plasmon, it is necessary to optimize them and more especially the gold film thickness as it controls the plasmon modes [51].

Here we show that gold nanocylinders grown on films with a well-controlled geometry represent an advanced plasmonic architecture for multispectral SERS sensing applications, characterized by an efficient response in a broad range of excitation wavelength from the visible to the near infrared. This type of substrates could provide a real added value in SERS and its application in the sensing field.

## 2. Materials and Methods 

### 2.1. Nanostructure Fabrication

**Figure 1:**
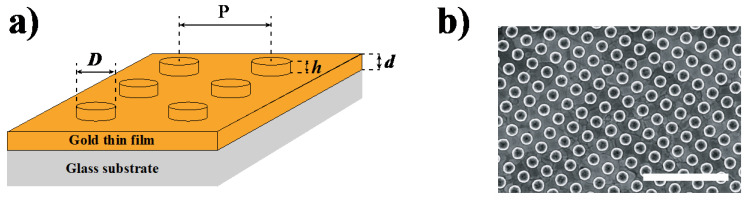
(**a**) Scheme of gold nanocylinders deposited on a gold thin film (*D*: nanocylinder diameter, *h*: nanocylinder height, *P*: grating period, *d*: film thickness); (**b**) SEM image of nanocylinders with a 250 nm diameter on a gold film (scale bar: 2 μm).

The plasmonic substrates are made of square arrays (period *P* of 400 nm) of gold nanocylinders on a flat gold thin film (Figure 1). The nanocylinder diameters (*D*) were varied from 80 to 250 nm (±5 nm) whereas the nanocylinder height, *h*, is fixed at 40 nm. Four different film thicknesses, *d*, were used: 20, 30, 40 and 50 nm ± 3 nm.

A gold film with a specific thickness is first evaporated on a glass substrate (commercial 0.7 mm thick glass slide from Lumtec technology, Taipei, Taiwan) using an evaporation process (Plassys, France). Then, using scanning electron microscopy (Pioneer, Raith, Dortmund, Germany) the arrays of gold nanocylinders were produced by electron beam lithography operating at 20 keV with a beam current of 15 pA. The electron beam is used to produce nanocylinder gratings with the same settings for all thicknesses on a film of polymethylmethacrylate (PMMA, Sigma Aldrich, Darmstadt, Germany purity: 99%, Mw = 97,000, 60 g/L diluted in MIBK, Dow Chemical, purity: 99.6%, thickness = 140 nm) deposited on the gold thin film by spin coating (300 rpm). The PMMA development was done for 60 s in a solution of 1:3 methyl isobutyl ketone:isopropanol (MIBK:IPA, analytical reagent grade Fisher Chemical, Paris, France, purity: 99.7%). In the final step, to produce the nanocylinders, 40 ± 3 nm of gold was deposited on the PMMA by thermal evaporation. The PMMA is then removed by a lift off process in acetone to obtain the gold nanostructures.

Four samples were then produced: one for each gold film thickness (20, 30, 40 and 50 nm) having 18 nanocylinder gratings (one grating for each diameter from 80 up to 250 nm). For each diameter, the grating has a size of 80 × 80 µm^2^. To verify the nanocylinder arrays, a scanning electron microscopy (Pioneer, Raith, Dortmund, Germany) was used.

### 2.2. Extinction Spectroscopy

Extinction spectra were performed using an XploRA Raman micro spectrometer (Horiba Scientifics) (Longjumeau, France) after the removal of the edge filters. A non-polarized white light illuminates the sample from the bottom (glass side). The light transmitted through the plasmonic substrate is collected with a low magnification (X10) microscope objective having a low numerical aperture (0.25) in order to avoid the scattered light. The extinction spectrum is calculated as the ratio between the transmitted light on the substrate with the nanostructures and a reference spectrum taken on the substrate without the nanocylinder (only the gold thin film).

The extinction spectra were measured with normal and angular incidences. For the angular extinction spectra, the substrate was tilted from 0° to 50° with a home-made set-up. 

### 2.3. Surface-Enhanced Raman Spectroscopy

Raman measurements were done using an XploRA spectrometer (Horiba Scientifics, Longjumeau, France) for the two excitation wavelengths at 532 and 638 nm, and a WITec alpha 300 R spectrometer for the 785 nm excitation wavelength. The samples where functionalized with 4-mercaptobenzoic acid (MBA, Sigma Aldrich, Darmstadt, Germany, purity: 90%). The samples have been immerged in a solution of 1 mM of MBA during one night to form a self-assembled monolayer at the gold surface. The SERS signal was recorded in backscattering configuration with an objective X100 having a numerical aperture of 0.9. For each wavelength, the laser power and the acquisition time were fixed (532 nm: 10 s and 5.6 mW; 638 nm: 10 s and 1.4 mW; 785 nm: 0.5 s and 20 mW) to be able to compare the SERS signal from the different plasmonic substrates. To compare the SERS efficiencies from one wavelength to another, we measured the Raman spectrum of a reference material. In our case, we used Si wafer with a crystal orientation of (100). To record the Si Raman signal, we used the following laser power and acquisition time: at 532 nm: 1 s and 5.6 mW; at 638 nm: 0.5 s and 1.4 mW; at 785 nm: 0.5 s and 20 mW. To normalize the SERS signal by the Si signal, we first calculated the intensity per laser power and per second for both signals and then we calculated the ratio I_SERS_/I_Si_. To take into account the fact that the penetration depth inside the Si is not the same for each wavelength, we calculate a correction factor that depends on the excitation wavelength. The details of the calculation of this factor is given in the SI (Figure S9).

## 3. Results and Discussion

**Figure 2:**
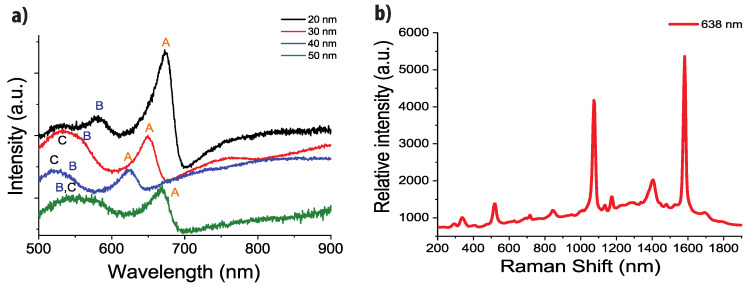
(**a**) Extinction spectra for nanocylinders with a diameter of 250 nm and for different gold film thicknesses (black spectrum: 20 nm, red spectrum: 30 nm, blue spectrum: 40 nm, green spectrum: 50 nm); (**b**) surface enhanced Raman scattering (SERS) spectra of MBA on gold nanocylinders with a diameter of 250 nm and a film thickness of 20 nm. Excitation wavelength: 638 nm.

Figure 2 shows the extinction spectra of the substrates as a function of the gold film thickness. Three surface plasmon bands, called A, B and C in Figure 2, can be observed: band A between 600 and 700 nm, band B around 550 nm and band C around 530 nm (See also the Figure S1).

All bands can be assigned to the excitation of propagative plasmon at the gold/glass interface through the diffraction orders of the nanocylinder grating: orders (0, ±1) and (±1, 0) for the band A and orders (±1, ±1) for the bands B and C. Their position is in fact nearly constant whatever the film thickness and the nanocylinder diameter. Shifts of a few tens of nanometers are observed due to the shift of the plasmon energy with the gold film thickness. More detailed analysis of the plasmon modes and their dependence on the film thickness can be found in ref. [47]. 

To determine the SERS efficiency of each substrate, we immersed the substrates in a probe molecule solution (1 mM of 4-mercaptobenzoic acid, MBA). Figure 2b shows the SERS spectrum of the MBA obtained for the excitation wavelength of 638 nm (For the other wavelengths 532 and 785 nm see Figure S2) and a gold film thickness of 20 nm. The SERS spectrum exhibits two mains bands at 1075 cm^−1^ and 1580 cm^−1^ assigned to vibration of the aromatic cycle (ring breathing mode) [55,56].

### 3.1. Influence of the Geometrical Parameters 

Let us first discuss the influence of the geometrical parameters of the plasmonic substrate on the relative SERS intensity.

**Figure 3:**
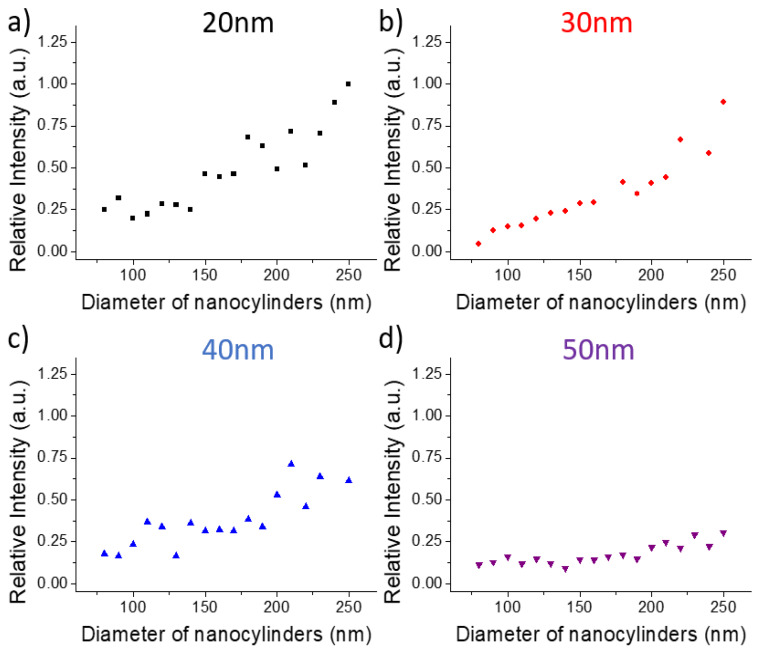
Evolution of the SERS intensities of the band located at 1580 cm^−1^ depending on the nanocylinder diameter and for the four film thicknesses: (**a**) 20, (**b**) 30, (**c**) 40, and (**d**) 50 nm. Excitation wavelength: 638 nm. Points size includes the error bars.

**Figure 4:**
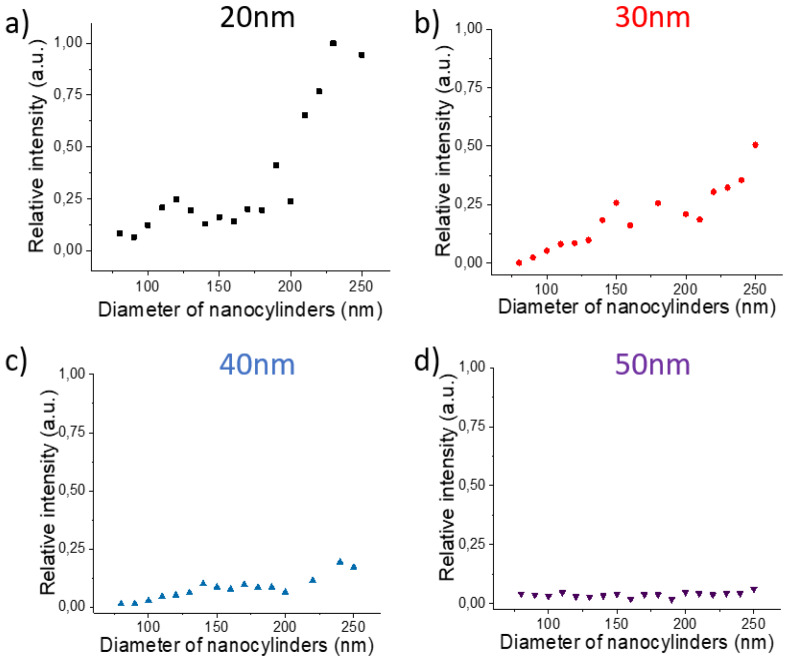
Evolution of the SERS intensities of the band located at 1580 cm^−1^ depending on the nanocylinder diameter and for the four film thicknesses: (**a**) 20, (**b**) 30, (**c**) 40, and (**d**) 50 nm. Excitation wavelength: 785 nm. Points size includes the error bars.

**Figure 5:**
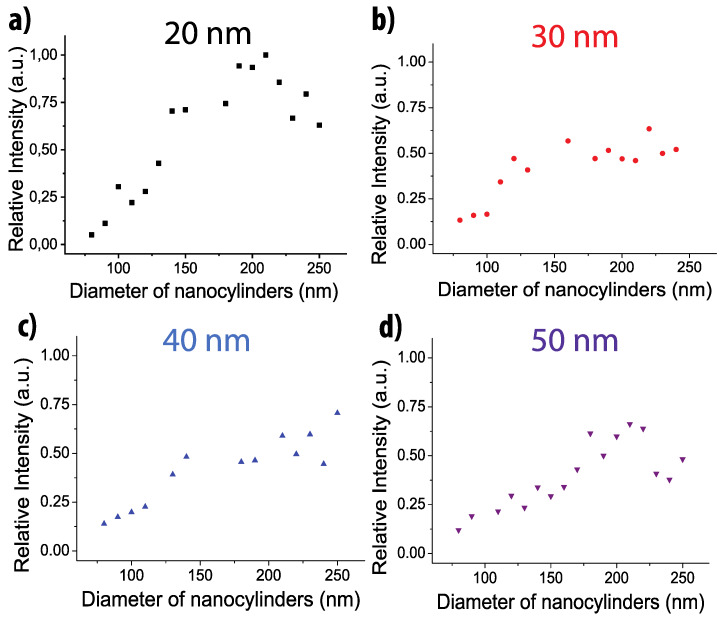
Evolution of the SERS intensities of the band located at 1580 cm^−1^ depending on the nanocylinder diameter and for the four film thicknesses: (**a**) 20, (**b**) 30, (**c**) 40, and (**d**) 50 nm. Excitation wavelength: 532 nm. Points size includes the error bars.

The SERS intensities for each diameter and each thickness were recorded on 50 points at the substrate surface and averaged. We then normalized these average values by the highest intensity measured for each wavelength. The results for the 1580 cm^−1^ band are shown on the Figure 3, Figure 4 and Figure 5 (results for 1080 cm^−1^ bands are presented on the Figures S3–S5).

For all wavelengths and all thicknesses, the SERS intensity increases with the diameter and reaches a maximum for the largest diameters (except at 532 nm for the thicknesses of 20 and 50 nm with a maximum for a diameter around 210 nm).

Let us now focus on the size of the nanocylinders and its potential influence on the SERS signal. Indeed, as the diameter increases, the gold surface of the nanocylinder increases inducing an increase of the number of adsorbed molecules and as a consequence a larger Raman signal. On a previous study, it has been demonstrated that for such plasmonic substrates, the SERS signal comes essentially from the edges of the nanocylinder [52] whereas the top and the gold between the nanocylinders have negligible contribution to the SERS signal. We have then simulated the variation of the surface of the edges of the nanocylinder and compared it with the variation of the SERS signal (Figures S6–S8). As the edge surface is proportional to the diameter (Surface = π *× D × h*), this variation is linear with the diameter has shown on the Figures S6–S8. One can notice that in most of the cases, the variation of the SERS signal nearly follows the linear edge surface variation for diameter lower than 200 nm. Thus, the SERS signal is directly related to the increase of the surface of the enhanced near-field that becomes larger for larger diameters. However, for diameters larger than 200 nm, the SERS signal increases more rapidly than the edge surface. The experimental curve slope is clearly modified and the faster increase of the SERS signal could be induced by a coupling effect between adjacent nanocylinders. As the grating period is kept constant to 400 nm, the gap between the nanocylinders decreases when the diameter increases. We could assume that the enhanced fields between two nanocylinders couple together for a gap lower than 200 nm. This observation is remarkable as for nanostructures on dielectric substrates, the gap distance to induce the near-field coupling should be lower than 50 nm [39,40]. In the case of gold nanostructures on gold film, the coupling occurs for largely higher gap.

One can also notice that the enhancement exhibits different behavior depending on the gold film thickness. For all wavelengths, the SERS signal is the highest for the lowest thickness (20 nm) and decreases when the thickness increases. Some specific behavior can be observed. At 638 nm, the SERS is similar for 20 and 30 nm and then decreases for 40 and 50 nm. At 785 nm, the SERS signal decreases quickly with the thickness. Finally, for 532 nm, the SERS signal is nearly constant for all thicknesses except for the 20 nm thickness where the SERS is maximum. Thus, for all excitation wavelengths, an optimized thickness (20 nm) can be identified and a decreasing signal with increasing thickness can be observed. Such behavior is due to the nature of the surface plasmon excited in SERS. Indeed, with such thicknesses and nanocylinder grating periods, we excite the propagative plasmon at the interface between the glass and the gold. Thus, when the thickness increases, the distance between the surface plasmon and the interface between air and gold increases inducing a lower efficiency of the enhancement at the substrate surface and then a lower SERS signal.

### 3.2. Influence of Excitation Wavelength on SERS Signal 

We also determined the influence of the excitation wavelength on the SERS efficiency.

**Figure 6:**
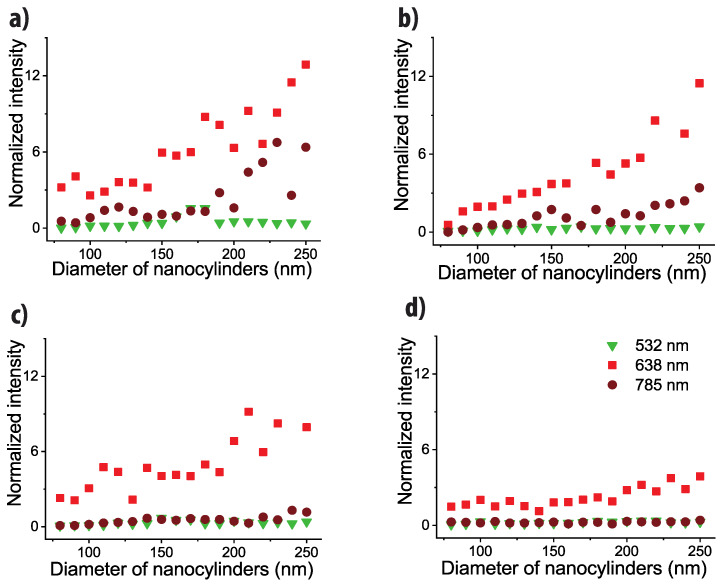
Evolution of the SERS intensities of the band located at 1580 cm^−1^ depending on the nanocylinder diameter and for the four film thicknesses: (**a**) 20, (**b**) 30, (**c**) 40, and (**d**) 50 nm. The SERS intensities have been normalized by the silicon Raman signal. Green triangles: excitation wavelength of 532 nm; Red squares: excitation wavelength of 638 nm; Brown circles: excitation wavelength of 785 nm.

We do not calculate precisely the absolute enhancement factor of our substrate, due to the difficulties of the approach and the potential uncertainties that could be induced by the process [57], but we can estimate it to be close to 10^7^ as already measured for similar SERS substrates [49,50]. However, in order to compare the SERS efficiency from one wavelength to another and to take into account the differentiate apparatus response at each wavelength, we normalized the SERS signal by one reference signal. To do so, we measured the Raman signal of a silicon crystal at each wavelength. Then we divided the SERS signal by the signal recorded for the band at 520 cm^−1^. Such an approach allows us to suppress the spectral response of the Raman spectrometer and to be able to compare the SERS signal from one wavelength to another (more details are given in SI9). The results are shown in Figure 6.

Regardless of the film thickness, the 638 nm excitation wavelength provides the best SERS signal and 532 nm provides the lowest one. At 785 nm, the SERS signal is comparable to the one obtained at 638 nm (slightly lower) for the thicknesses of 20 and 30 nm but it becomes very low and comparable to the one obtained at 532 nm for the thicknesses of 40 and 50 nm.

Thus, even if we are able to get some SERS signal at all wavelengths and on the whole visible range with an individual substrate, it means that there is also an optimization on the most efficient excitation wavelength. Regarding the extinction spectra (Figure 2), and as already demonstrated for the nanostructures on dielectric substrate [37], we can expect that the highest signal is reached for a spectral range where some plasmon bands are observable. As a consequence, we should have a high signal at 638 and 532 nm and not at 785 nm, which is in contradiction with our experimental results.

**Figure 7:**
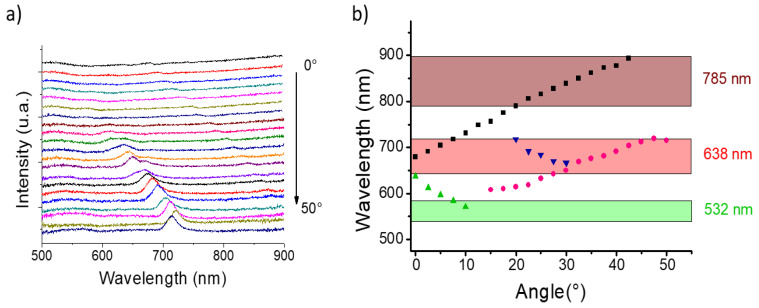
(**a**) Extinction spectra for our plasmonic substrate (nanocylinder diameter: 140 nm and thickness: 30 nm) depending on the incident angle (from 0 to 50°, step of 2.5°). (**b**) Evolution of the position of the different plasmon bands depending on the incident angle (each symbol corresponds to the position of one band on the spectra shown in (a) and each color shows the evolution of one plasmon mode). The green, red and brown rectangles correspond to the spectral ranges between the excitation wavelength and the 1580 cm^−1^ Raman band (532–581, 638–710, and 785–896 nm respectively).

In fact, such a contradiction can be explained by the plasmonic properties of the substrate as shown in the following. First, we excite the substrate with a microscope objective having a high numerical aperture (NA = 0.9) and thus the excitation cone includes a wide range of incident angles (from 0° up to 64°). Second, as the excited plasmon is a propagative one, its position is not constant but depends on the excitation angle as shown in Figure 7, through the modification of the grating constant with the angle. 

One can observe some modes splitting and some red or blue shifts with the angle (Figure 7). All these modes can potentially be excited through the objective cones and be SERS active in excitation or in collection as demonstrated in [51]. The position of the plasmon bands depending on the incidence angle are reported on the Figure 7b. The three spectral ranges between the excitation wavelength, λ_0_ (532, 638 and 785 nm), and the Raman scattering, λ_R_ (581, 710 and 896 nm, respectively, for the plasmon band at 1580 cm^−1^, are also indicated. One can notice that for the two ranges at 638 and 785 nm, several plasmons will be excited whereas a very few contribute on the 532 spectral range. As the SERS signal is directly related with the excitation of plasmon modes inside or close to the λ_0_-λ_R_ spectral range [37], the evolution of the SERS signal with the excitation wavelength can be explained by the overlap between the plasmon modes and the spectral ranges. A clear effect of the number of excited modes on the SERS efficiency can be observed: higher number of modes, higher SERS efficiency. Moreover, one can notice that at 532 nm, the λ_0_-λ_R_ spectral range overlaps more plasmon bands for the band at 1580 cm^−1^ (532–581 nm) than at 1080 cm^−1^ (532–564 nm). This could explain why the former band is more intense than the latter as the reradiation effect (enhancement at the Raman scattering wavelength) is more favored at 1580 cm^−1^ than at 1080 cm^−1^ (Figure S2). For 785 nm (Figure S2), the opposite effect will occur as more plasmon bands will favor the reradiation at 858 nm (1080 cm^−1^) than at 896 nm (1580 cm^−1^). By controlling the position of the plasmon modes and having a larger tunability on the visible range, we assume that we could provide potentially a nearly constant enhancement for a wide range of excitation wavelength.

## 4. Conclusions

We presented results on the SERS efficiency of plasmonic substrates made of gold nanocylinders on a gold film and discussed the influence of the geometrical parameters (film thickness and nanocylinder diameter) on the SERS signal of MBA in order to optimize it. As we only measure the SERS signal for a single molecule, we only probe some information on the electromagnetic enhancement factor without taking into account any potential chemical effect. Thus, our study is exploratory and further experiments should be provided with other analytes as probe molecules (methylene blue, BPE) or molecules of interest (DNA strands, proteins, pollutants, etc.).

We demonstrated that when the diameter of the nanocylinder increases, we reach a higher SERS signal whatever the thickness of the film is. The increase of the diameter of the nanocylinders has one main consequence: the gap between the nanostructures is reduced allowing for a coupling between them. Thanks to the gold film below the gold nanocylinders, this coupling occurs for gap lower than 200 nm generating more efficiency on SERS. Thus such effect takes place on a long range compared to nanocylinders on dielectric substrates. It is an advantage, as it is easier to produce and then to control.

When the gold film thickness decreases, the signal increases to attend its optimum for the lowest thickness. Generally, when the thickness changes only the intensity of the signal is affected and not the signal evolution.

Even if we conclude that 638 nm is the excitation wavelength providing the highest signal, such a substrate has the ability to provide SERS signals at several wavelengths on nearly the whole visible range (from 500 up to 800 nm). This is due to potential excitation of plasmons on a wide spectral range, as revealed by the angular study, and whose positions coincide with the excitation wavelength and the Raman scattering. This is a clear advantage of such substrates. By exploiting their specific plasmonic properties and by optimizing their geometrical parameters (film thickness, array period, nanostructure shape and size) we could design a multispectral SERS substrate providing a large SERS efficiency on a wide spectral range and which could be used at several wavelengths.

## Supplementary Materials

The following are available online at https://www.mdpi.com/2079-4991/10/5/927/s1, Figure S1: Extinction spectra for the four film thicknesses; Figure S2: SERS spectra of MBA on gold nanocylinders; Figure S3: Evolution of the SERS intensities of the band located at 1080 cm^−1^ depending on the nanocylinder diameter and for the four film thicknesses. Excitation wavelength: 633 nm; Figure S4: Evolution of the SERS intensities of the band located at 1080 cm^−1^ depending on the nanocylinder diameter and for the four film thicknesses. Excitation wavelength: 785 nm; Figure S5: Evolution of the SERS intensities of the band located at 1080 cm^−1^ depending on the nanocylinder diameter and for the four film thicknesses. Excitation wavelength: 532 nm; Figure S6: Comparison of the variation of the surface of the nanocylinder edges and the evolution of the SERS intensities of the band located at 1580 cm^−1^ depending on the nanocylinder diameter and for the four film thicknesses. Excitation wavelength: 638 nm; Figure S7: Comparison of the variation of the surface of the nanocylinder edges and the evolution of the SERS intensities of the band located at 1580 cm^−1^ depending on the nanocylinder diameter and for the four film thicknesses. Excitation wavelength: 785 nm; Figure S8: Comparison of the variation of the surface of the nanocylinder edges and the evolution of the SERS intensities of the band located at 1580 cm^−1^ depending on the nanocylinder diameter and for the four film thicknesses. Excitation wavelength: 532 nm; Figure S9: Normalization of the SERS intensity by the Si Raman intensity.
