# Supplementary material for: Gold Nanocylinders on Gold Film as a Multi-Spectral SERS Substrate
Source: Nanomaterials (Basel). 2020 May 11;10(5):927. doi: 10.3390/nano10050927 (PMC7279415; doi:10.3390/nano10050927)
Supplement: Supplementary file 1 [file nanomaterials-10-00927-s001.pdf]

nanomaterials

MDPI

Article

Gold nanocylinders on gold film as a multi-spectral SERS substrate

Wafa Safar[1], Médéric Lequeux[2], Jeanne Solard[3], Alexis P.A. Fischer[3], Nordin Felidj[4], Pietro Giuseppe Gucciardi[5], Mathieu Edely[1], Marc Lamy de la Chapelle[1*]

1 Institut des Molécules et Matériaux du Mans (IMMM - UMR CNRS 6283), Université du Mans, Avenue Olivier Messiaen, 72085 Le Mans cedex 9, France.
2 Université Paris 13, Laboratoire CSPBAT, CNRS, (UMR 7244), 74 rue Marcel Cachin, 93017 Bobigny, France.
3 Université Paris 13, Laboratoire de Physique des Lasers, CNRS, (UMR 7538), 99 av. JB Clément, 93450 Villetaneuse, France.
4 Université de Paris, ITODYS, CNRS, UMR 7086, 15 rue J-A de Baïf, F-75013 Paris, France.
5 CNR IPCF, Istituto per i Processi Chimico-Fisici, Viale F. Stagno D'Alcontres 37, I-98158 Messina, Italy.
* Institut des Molécules et Matériaux du Mans (IMMM - UMR CNRS 6283), Université du Mans, Avenue Olivier Messiaen, 72085 Le Mans cedex 9, France. E-mail: marc.lamydelachapelle@univ-lemans.fr

Received: date; Accepted: date; Published: date

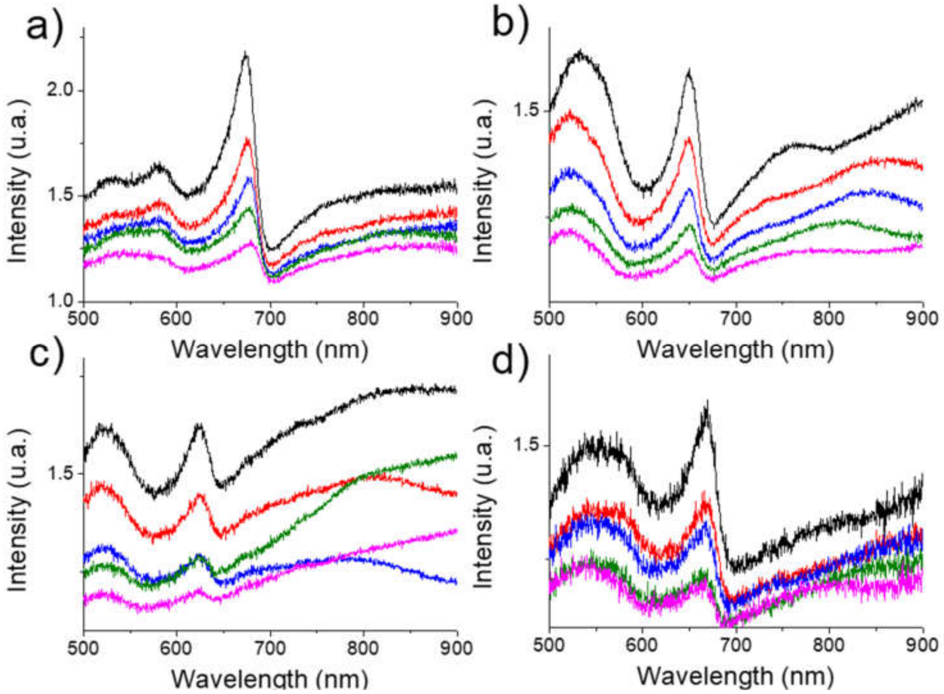

Figure SI1: Extinction spectra for the four film thicknesses: a) 20 nm, b) 30 nm, c) 40 nm, d) 50 nm and different nanocylinder diameters (black spectrum: 250 nm, red spectrum: 230 nm, blue spectrum: 210 nm, green spectrum: 190 nm, purple spectrum: 170 nm).

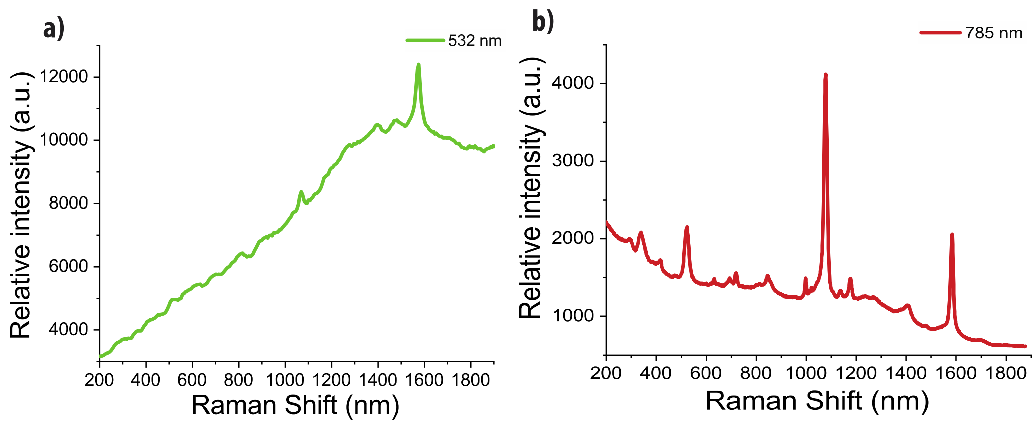

Figure SI2: SERS spectra of MBA on gold nanocylinders with a diameter of 250 nm and a film thickness of 20 nm at the excitation wavelengths of (a) 532 and (b) 785 nm.

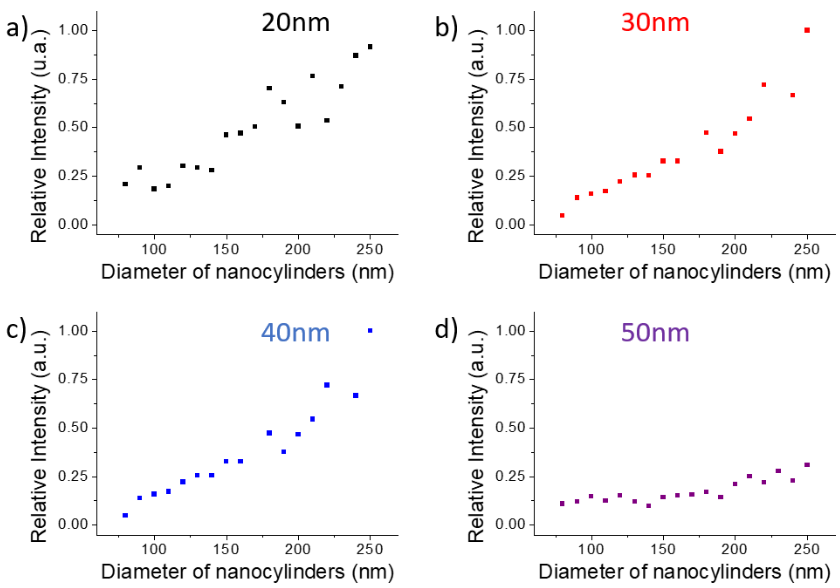

Figure SI3: Evolution of the SERS intensities of the band located at 1080 cm^{-1} depending on the nanocylinder diameter and for the four film thicknesses: a) 20 nm, b) 30 nm, c) 40 nm, d) 50 nm. Excitation wavelength: 638 nm. Points size include the error bars.

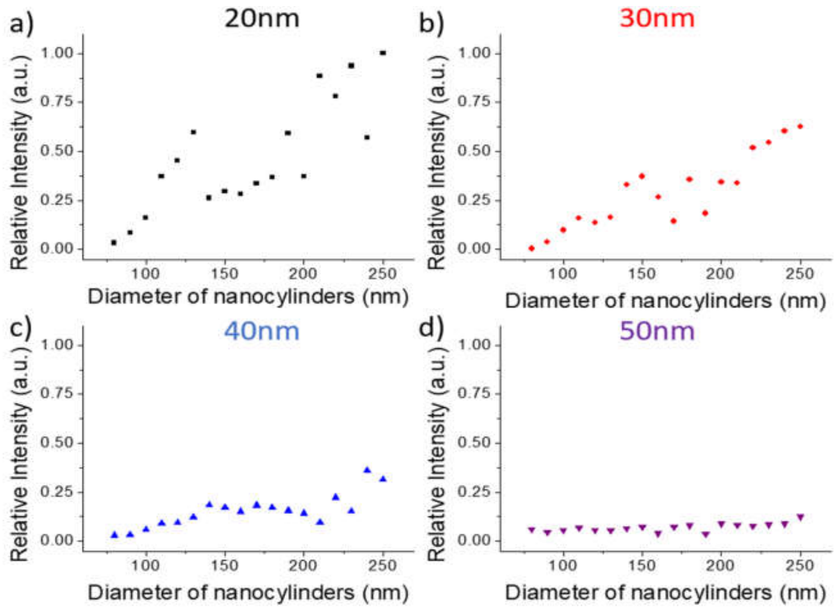

Figure SI4: Evolution of the SERS intensities of the band located at 1080 cm^{-1} depending on the nanocylinder diameter and for the four film thicknesses: a) 20 nm, b) 30 nm, c) 40 nm, d) 50 nm. Excitation wavelength: 785 nm. Points size include the error bars.

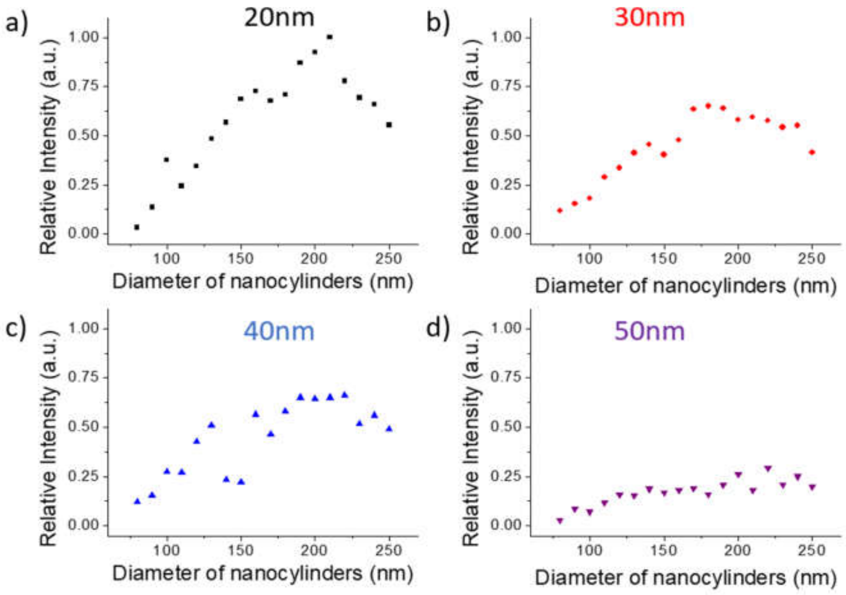

Figure SI5: Evolution of the SERS intensities of the band located at 1080 cm^{-1} depending on the nanocylinder diameter and for the four film thicknesses: a) 20 nm, b) 30 nm, c) 40 nm, d) 50 nm. Excitation wavelength: 532 nm. Points size include the error bars.

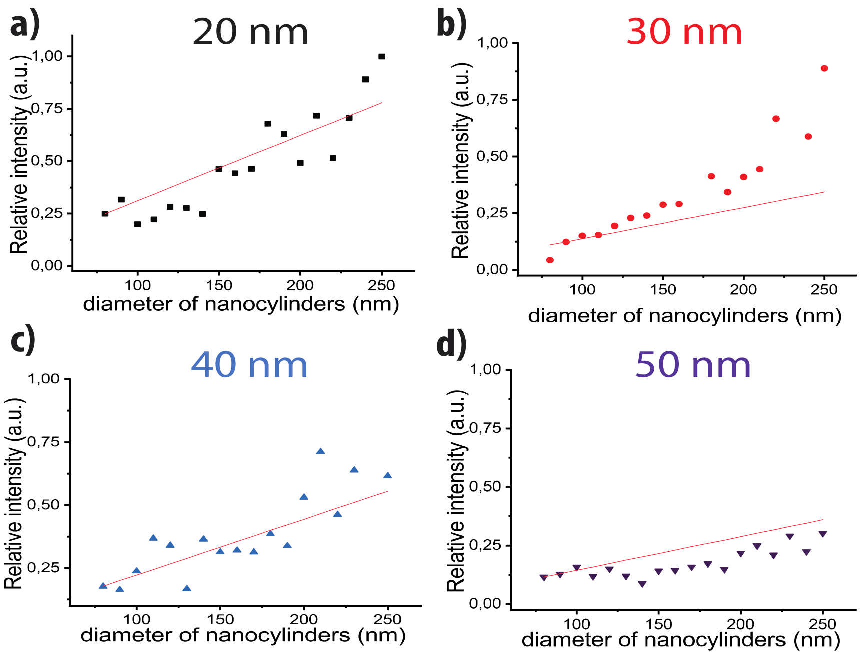

Figure SI6: Comparison of the variation of the surface of the nanocylinder edges (red lines) and the evolution of the SERS intensities of the band located at 1580 cm^{-1} depending on the nanocylinder diameter and for the four film thicknesses: a) 20 nm, b) 30 nm, c) 40 nm, d) 50 nm. Excitation wavelength: 638 nm.

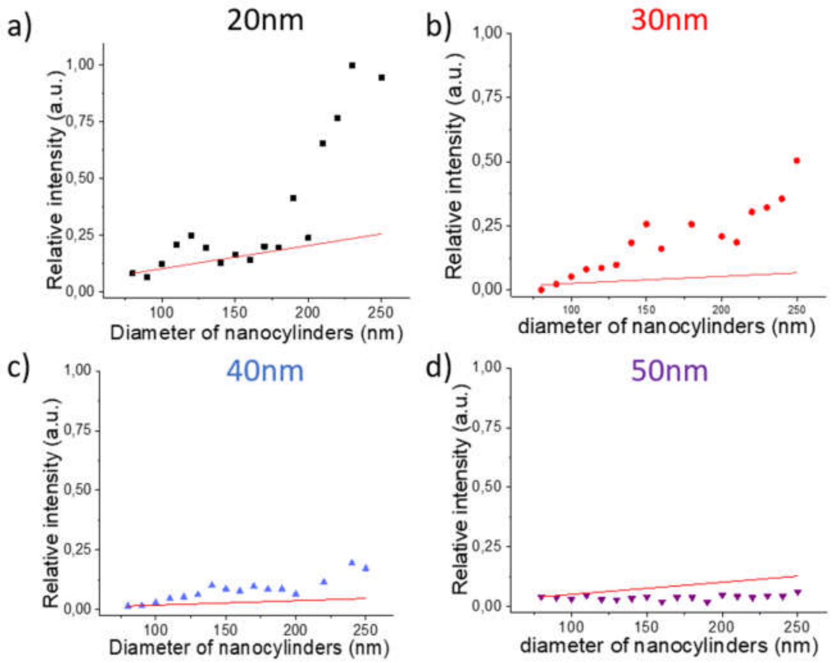

Figure SI7: Comparison of the variation of the surface of the nanocylinder edges (red lines) and the evolution of the SERS intensities of the band located at 1580 cm^{-1} depending on the nanocylinder diameter and for the four film thicknesses: a) 20 nm, b) 30 nm, c) 40 nm, d) 50 nm. Excitation wavelength: 785 nm.

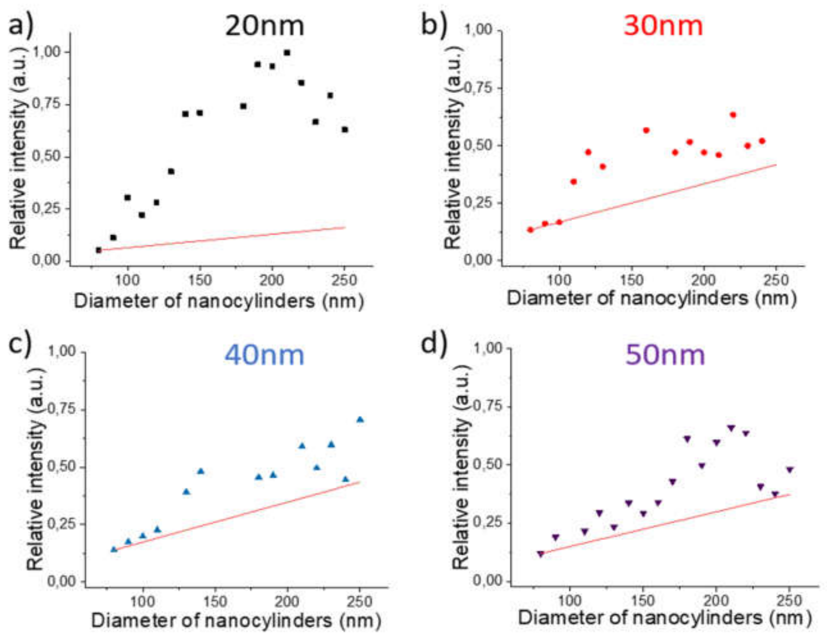

Figure SI8: Comparison of the variation of the surface of the nanocylinder edges (red lines) and the evolution of the SERS intensities of the band located at 1580 cm^{-1} depending on the nanocylinder diameter and for the four film thicknesses: a) 20 nm, b) 30 nm, c) 40 nm, d) 50 nm. Excitation wavelength: 532 nm.

SI9: Normalization of the SERS intensity by the Si Raman intensity

The Raman intensity of the Si, $I_{Si}(\lambda)$, depends on the volume excited by the laser. Such volume depends on the excitation wavelength, λ, and the volume probes by the laser is then different from one wavelength to another.

Thus, as $I_{Si}(\lambda)$ is proportional to the $V_{Si}(\lambda)$, it has to be normalized by the $V_{Si}(\lambda)$ to be comparable from one wavelength to another.

$$I_{Si\_Norm}(\lambda) = \frac{I_{Si}(\lambda)}{V_{Si}(\lambda)}$$

One can assume that the confocal volume has an ellipsoidal shape as shown on the figure below, with r_{xy}, the radius in the sample surface and r_z, the half axis perpendicular of the sample surface.

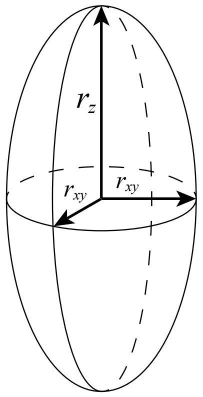

In diffraction limited optics, $r_{xy} = \frac{0.61\lambda}{NA}$ and $r_z = \frac{2n\lambda}{NA^2}$, with *n*, the refractive index and *NA*, the objective numerical aperture.

The volume, V_{Si}, probes by the laser inside the material is the half of the ellipsoid volume and can be calculated as follow: $V_{Si} = \frac{2}{3}\pi r_{xy}^2 r_z$.

In the same way, to be comparable from one wavelength to another, the SERS intensities have also to be corrected by the surface of collection $S_{SERS} = \pi r_{xy}^2$ (the volume is not applicable as the SERS is only a surface process).

$$I_{SERS\_Norm}(\lambda) = \frac{I_{SERS}(\lambda)}{S_{SERS}(\lambda)}$$

To compare the SERS intensity between the different wavelength, we then divided the $I_{SERS\_Norm}(\lambda)$ by the $I_{Si\_Norm}(\lambda)$ to remove the apparatus response that is wavelength dependent.

$$\frac{I_{SERS\_Norm}(\lambda)}{I_{Si\_Norm}(\lambda)} = \frac{I_{SERS}(\lambda)}{S_{SERS}(\lambda)}\frac{V_{Si}(\lambda)}{I_{Si}(\lambda)} = \frac{I_{SERS}(\lambda)}{I_{Si}(\lambda)}\frac{2}{3}r_z(\lambda) \propto \frac{I_{SERS}(\lambda)}{I_{Si}(\lambda)}\lambda$$

The $\frac{I_{SERS\_Norm}(\lambda)}{I_{Si\_Norm}(\lambda)}$ is then proportional the $\frac{I_{SERS}(\lambda)}{I_{Si}(\lambda)}$ ratio and to λ. The $\frac{I_{SERS}(\lambda)}{I_{Si}(\lambda)}$ is then relevant to determine the relative enhancement factor from one wavelength to another but should be corrected by a factor to take into account the proportionality to λ.

Thus at 532 nm, the $\frac{I_{SERS\_Norm}(\lambda)}{I_{Si\_Norm}(\lambda)}$ is overestimated by the $\frac{I_{SERS}(\lambda)}{I_{Si}(\lambda)}$ as the excitation wavelength is lower whereas at 785 nm, the $\frac{I_{SERS\_Norm}(\lambda)}{I_{Si\_Norm}(\lambda)}$ is underestimated as the excitation wavelength is larger. If we fixed the reference $\frac{I_{SERS\_Norm}(\lambda)}{I_{Si\_Norm}(\lambda)}$ for the wavelength at 638 nm (assuming that λ =1 in the equation $\frac{I_{SERS\_Norm}(\lambda)}{I_{Si\_Norm}(\lambda)} \propto \frac{I_{SERS}(\lambda)}{I_{Si}(\lambda)}\lambda$), the $\frac{I_{SERS}(\lambda)}{I_{Si}(\lambda)}$ ratio has to be multiplied by $\frac{638}{532} = 0{,}834$ at 532 nm and by $\frac{785}{638} = 1{,}23$ at 785 nm to be comparable with the $\frac{I_{SERS}(\lambda)}{I_{Si}(\lambda)}$ at 638 nm.

We then corrected the $\frac{I_{SERS}(\lambda)}{I_{Si}(\lambda)}$ ratio in the figure 6 to remove the wavelength dependence of the $I_{Si}(\lambda)$.
